# 555. Early Outcomes of Penicillin and Cephalosporin Allergy Assessment Tool

**DOI:** 10.1093/ofid/ofac492.608

**Published:** 2022-12-15

**Authors:** Laura E Norton, Lily Y Grothe, Meredith B Oliver, Beth K Thielen

**Affiliations:** University of Minnesota, Minneapolis, Minnesota; University of Minnesota, Minneapolis, Minnesota; M Health Fairview Masonic Children’s Hospital, Minneapolis, Minnesota; University of Minnesota, Minneapolis, Minnesota

## Abstract

**Background:**

Penicillin allergy is reported in approximately 10% of the US population and is one of the most frequent medication allergies in children. Penicillin allergy labels are associated with more expensive care and adverse outcomes, such as MRSA and C. Diff infection. Studies suggest up to 90% of these patients could safely tolerate this class of drugs. Our team at M Health Fairview University of Minnesota Masonic Children’s Hospital (UMMCH) implemented an allergy assessment tool for inpatient use in January of 2020 to create an effective and accessible path for delabeling pediatric patients with penicillin and cephalosporin allergies.

**Methods:**

Our pharmacists conducted the allergy assessment on inpatient pediatric patients with penicillin/cephalosporin allergies. Those deemed low to moderate risk for having a true IgE-mediated allergy by our assessment were recommended for outpatient allergy testing. Those deemed no risk were recommended for immediate delabeling. We performed manual chart reviews to describe pediatric patient risk stratification, allergy label status after assessment, outpatient allergy follow-up, and status and results of allergy testing.

**Results:**

From January, 2020 to present, 140 patients received a pharmacist allergy assessment. During the 2020 calendar year, 51 allergy assessments were performed on 49 patients. 58 allergies were recorded, with penicillins as the most common (62%), followed by cephalosporins (24%), and penicillin combination drugs (14%). 1.9% of allergies were high risk, 68.8% moderate, 15.7% low, 9.8% no risk, and 3.8% difficult to determine. Of 42 patients deemed low to moderate risk, 8 (17%) were seen by an outpatient allergist. Of these, 5 proceeded with allergy testing and were found to be not allergic. Out of 58 allergy labels, 9 (15.5%) were removed, 5 after allergy testing, 2 immediately due to no risk, and 2 after further history review.

**Conclusion:**

Our preliminary data show allergy assessments can inform pediatric allergy risk prior to outpatient allergy testing and assist with imminent delabeling. Next steps include analysis of our 2021 assessments as well as patient caregiver surveys to assess perception of the allergy assessment process and to elucidate on barriers to testing/delabeling.

**Disclosures:**

**Beth K. Thielen, MD, PhD**, Horizon: Advisor/Consultant|Horizon: Honoraria|Merck: Grant/Research Support.

